# Paraoxonase Activity and Expression Is Modulated by Therapeutics in Experimental Rat Nonalcoholic Fatty Liver Disease

**DOI:** 10.1155/2012/265305

**Published:** 2012-03-27

**Authors:** O. Hussein, J. Zidan, K. Abu Jabal, I. Shams, S. Szvalb, M. Grozovski, I. Bersudsky, R. Karry, M. Aviram

**Affiliations:** ^1^Internal Medicine Department A, Ziv Medical Center, Safed 13100, Israel; ^2^Faculty of Medicine, Bar-Ilan University, 13100 Safed, Israel; ^3^Oncology Unit, Ziv Medical Center, Safed 13100, Israel; ^4^Pathology Department, Ziv Medical Center, Safed 13100, Israel; ^5^Biotechnology Department, Ort Braude College of Engeneering, Karmiel 21982, Israel; ^6^The Lipid Research Laboratory, Technion Faculty of Medicine, The Rappaport Family Institute for Research in the Medical Sciences and Rambam Medical Center, Haifa 31096, Israel

## Abstract

*Objective*. The objective of the present study is to investigate the effect of rosiglitazone, metformin, ezetimibe, and valsartan (alone or in combinations) on paraoxonase (PON) activity and PON-mRNA expression in nonalcoholic fatty liver disease (NAFLD). *Methods*. 54 Male Sprague–Dawley rats were divided to 9 groups: chow diet group (15 weeks); methionine-choline-deficient diet (MCDD) group (15 weeks); MCDD-treated groups for the last 6 weeks with either metformin (M), rosiglitazone (R), metformin plus rosiglitazone (M+R), ezetimibe (E), valsartan (V), or a combination of R+M+V or of R+M+V+E for a total period of 15 weeks. *Results*. PON activities in serum and liver were decreased in MCDD rats. PON activity in serum increased significantly in all treatment groups. PON activity in liver was also increased significantly, except only in groups R, E, V, R+M+V, and R+M+V+E. Liver PON3 mRNA expression increased significantly in groups R+M, E, V, R+M+V, and R+M+V+E whereas liver PON2 mRNA expression increased significantly in MCDD, R+M, E, V, R+M+V, and R+M+V+E. *Conclusions*. PON activities in serum and liver were decreased in NAFLD. Treatment with insulin sensitizers, ezetimibe, and valsartan increased PON activity and reduced oxidative stress both in serum and liver.

## 1. Background

Paraoxonase (PON) aryldialkylphosphatase is an ester hydrolase that catalyzes the hydrolysis of some xenobiotics, such as organophosphates, unsaturated aliphatic esters, aromatic carboxylic esters and, possibly, carbamates [1]. The paraoxonase gene family contains at least three members, PON1, PON2, and PON3, which are located on chromosome 7q21.3–22.1 [2–4]. PON1 and PON3 mRNA are predominantly expressed in liver, whereas PON2 mRNA is found in different tissues [5] including human endothelial and aortic smooth muscle cells [6]. The enzymes PON1 and PON3 are circulating in serum and tightly bound with HDL in serum, and several studies suggest that it is this association that contributes to the protection conferred by HDL against LDL oxidation [7, 8]. PON2 is cell associated and is not circulating in the serum [6]. PON2 has been shown to reduce reactive oxygen species (ROS) in HeLa cells, reverse the oxidation of oxidized low-density lipoprotein, and inhibit the ability of oxidized low-density lipoprotein to induce monocyte chemotaxis [6], which can contribute to its antiatherogenic properties.

Serum PON1 is synthesized mainly in the liver. The gene expression has been observed only in the liver [9, 10]. Arylesterase and PON1 activities have been shown to be functions of a single enzyme [11]. Lipid peroxidation products are increased [12, 13] and levels of endogenous antioxidants are decreased in patients with nonalcoholic liver disease (NAFLD) [14]. Chronic exposure to increased levels of oxidative stress may result in an excess of ROS within the hepatocytes which can contribute to the deterioration from NAFLD to non-alcoholic steatohepatitis (NASH). Liver antioxidant enzyme activities Cu/Zn-superoxide dismutase (SOD), glutathione peroxidase (GSHPx), and catalase were high in patients with NAFLD, but with normal thiobarbituric acid-reactive substance, which is a measure of the oxidative end product malonyl aldehyde (MDA) level [15]. Thus, in NAFLD, the increased antioxidant enzymes activities contained the oxidative stress and prevented the increment in MDA levels. Serum nitric oxide, SOD, GSHPx and PON1 activities [16], CuZn-SOD and catalase activities [17], and thiol levels [16, 18] were low in patients with NASH and incapable to compensate for oxidative stress. Increased lipid peroxidation and ROS in NAFLD consume antioxidant vitamins and can inactivate PON [19]. Decreased antioxidative defense may increase hepatocyte susceptibility to injury leading to aggravate NAFLD to NASH.

Decreased serum PON1 activity in NASH patients can be secondary to increased levels of proinflammatory cytokines such as interleukin-1 and TNF-*α* which downregulated mRNA expression of PON1 in HepG2 cells [20]. Decrease in liver microsomal PON1 activity is related to lipid peroxidation and liver injury in rats with CCl4-induced cirrhosis [21]. Decreased PON1 activity in sera of patients with chronic liver disease was suggested to be related to the degree of liver damage and not to allelic or genotypic differences [22]. On the contrary, in a recent study, there was no statistically significant correlation between degree of liver damage (grade and stage of NASH) from one side and between serum PON1 and MDA levels from the other side [23]. PON activity decreased significantly in the livers of Sprague-Dawley rats with experimental NAFLD induced by MCDD alone or enriched with olive oil, butter fat, or fish oil. The most prominent decrease in paraoxonase activity of 67.8% was observed in rats on MCDD enriched with olive oil [24]. The oxidative stress in the liver of this group was also higher than other groups on MCDD. As was previously reported by our group, MCDD rat group increased significantly the liver weight/rat weight ratio by 68%, hepatic triglyceride content by 1263%, and hepatic cholesterol content by 245% compared with the group on chow diet. In the group on chow diet (the control group), these parameters were 0.025 ± 0.003, 1.6 ± 0.3 mol/g, and 0.5 ± 0.0 mol/g, respectively. The MCDD group showed massive fatty infiltration, predominantly macrovesicular. There was mild ballooning degeneration and pericellular fibrosis [25]. Liver weight/body weight ratio in MCDD groups related to the ratio in chow group, liver, and serum triglyceride and cholesterol levels, serum alanine transaminase, fat infiltration in the liver and features of steatohepatitis were reported previously [25].

The key objectives of the present study are to investigate the effect of insulin sensitizers (rosiglitazone and metformin), ezetimibe, and valsartan (each alone or in combinations) on plasma PON activity and PON-mRNA expression in the liver and the potential protective role in NAFLD. To explain the paradox of increased oxidative stress with decreased PON activity in the MCDD rat group, we conducted this study.

## 2. Animal and Protocol

Male Sprague-Dawley rats (Harlan Laboratories Limited; Jerusalem, Israel) weighting 200–280 grams were studied. Rats were housed in regular cages situated in an animal room at 22°C. The rats before the beginning of the study were maintained on standard rat chow diet (Koffolk, Tel Aviv, Israel) and were given tap water to drink ad libitum. All animal studies were conducted according to the regulations for the use and care of experimental animals and treatment groups. The researchers were authorized to conduct the experiments after a formal training. The experiments were done in an authorized animal housing and laboratory systems.

Fatty liver was induced in rats fed by methionine-choline-deficient diet (MCDD TD.90262, Harlan Teklad, MadisonWI) for 9 weeks [26]. The constituents of MCDD as percent by weight were protein 14.9, carbohydrate 64.3, and fat 10.0. The rats were randomly divided to nine groups, 6 rats in each group. Group 1 served as control group and was maintained on standard chow diet for 15 weeks (control). Group 2 (MCDD group) was given only MCDD for 15 weeks. The following groups (3–9) were on MCDD but were treated during weeks 9–15 with various pharmaceutical interventions. Group 3 was fed MCDD with rosiglitazone (3 mg/kg), Group 4 was fed MCDD with metformin (200 mg/kg), Group 5 was fed MCDD with metformin + rosiglitazone, Group 6 was fed MCDD with ezetimibe (2 mg/kg), Group 7 was fed MCDD with valsartan (2 mg/kg), Group 8 was fed MCDD with metformin + rosiglitazone + valsartan, and Group 9 was fed MCDD with metformin + rosiglitazone + valsartan + ezetimibe. The dosage was selected for each intervention based on the results of previous studies using these agents in various liver diseases [27–30]. The dosage of the drugs in the combined treatments was the same as that in the groups treated with a single agent to maximize the combined effect. The standard rat chow diet contains 21.9% protein, 4.5% fat, 41% starch, 5% sugar, and 3.7% crude fibers. Diets were supplied in pellets form. The medications were given with food and in drinking water. All the drugs are water soluble and there was no need for additional solvents. The drugs were monitored daily and the formula was prepared and supplied by the local pharmacy of the Ziv Medical Center, Safed, Israel and not by pharmaceutical companies. The companies that produced the drugs were GlaxoSmithKline (rosiglitazone), Dexxon (metformin), MSD (ezetimibe), and Novartis (valsartan). The animals were kept at 37°C for 30 minutes before measurements, that were reported in previous publication, [25] were taken. Blood samples were taken by cardiac puncture. Rats were then sacrificed and liver and plasma were examined.

Malondialdehyde (MDA) levels were analyzed by the thiobarbituric acid reactive substances assay, which measures malondialdehyde equivalent [31].

Serum paraoxonase activity was determined by an adaptation of the spectrophotometric method of Furlong et al. [32]. Aliquots (10 *μ*L) of diluted (1 : 5) serum were placed in microliter plate wells in triplicate; the reaction was initiated by adding 190 *μ*L of the substrate (1.2 mmol/L paraoxon in 0.26 mmol/LTris-HCL, pH 8.5, 25 mmol/L CaCl2, and 0.5 mol/L Nacl). After mixing, the plate was read immediately at 450 nm to establish zero time values. Readings were repeated at 2 min interval for 10 min. Nonenzymatic hydrolysis of paraoxon was subtracted from the total rate of hydrolysis. The enzyme activity was calculated from the linear portion of the plot using the molar extinction for p-nitrophenol (17,100 mol/L). One unit of paraoxonase1 activity equals 1 *μ*mol/L·min of released p-nitrophenol.

PON arylesterase activity was measured by using phenylacetate as substrate. Nonenzymatic hydrolysis of phenylacetate was not observed. The reaction took place in Tris buffer (50 mM Tris-HCl, pH 8, 1 mM CaCl_2_) and was initiated by adding the substrate. Absorbance (*λ* = 270 nm) was measured at zero time and after 2 min. The enzymatic activity is expressed as micromoles of hydrolyzed phenylacetate.min^−1^·mL^−1^ (U/mL) or per mg of liver tissue [33].

Liver PON2 and PON3 mRNA expression by semiquantitative reverse transcriptase-polymerase chain reaction (RT-PCR): total RNA from rat livers was extracted with Tri-reagent (Sigma-Aldrich, Israel). cDNA was generated from 5 *μ*g of total RNA using 0.5 *μ*g random primers (Sigma-Genosys, Israel). Reverse transcriptase products were subjected to PCR amplification with Ready Mix PCR Master Mix (ABgene, Epsom, Surrey, UK). The amplification conditions were denaturation at 94°C for 15 s, annealing at 57°C for 20 s, and extension at 70°C for 15 s during 30 cycles for PON2 and PON3 [34]. The cDNA products were separated on 2% agarose gel with ethidium bromide. Densitometry was performed using Tina 2.0 g software (Fujifilm, Stockholm, Sweden).

## 3. Statistical Analysis

Results were expressed as mean ± standard deviation. ANOVA *Tukey's post hoc test *was used to examine differences between means. *P* < 0.05 was considered significant.

## 4. Results

**Figure 1:**
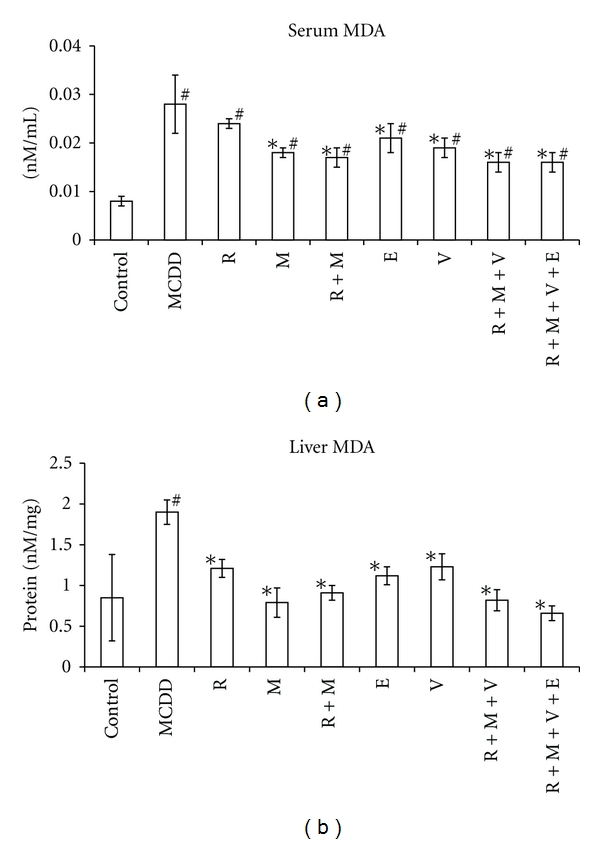
Malondialdehyde (MDA) in sera (a) and livers (b) in rat experimental fatty liver model. Fatty liver in Sprague-Dawley rats was induced by methionine-choline-deficient diet (MCDD) for 9 weeks. Rats were treated for other 6 weeks with rosiglitazone (R); metformin (M); rosiglitazone + metformin combination (R+M); ezetimibe (E); valsartan (V); rosiglitazone + metformin + valsartan in combination (R+M+V), or rosiglitazone + metformin + valsartan + ezetimibe (R+M+V+E). “^#^” indicates *P* < 0.05 versus control group (fed by standard rat chow). “*” indicates *P* < 0.05 versus MCDD group.

**Figure 2:**
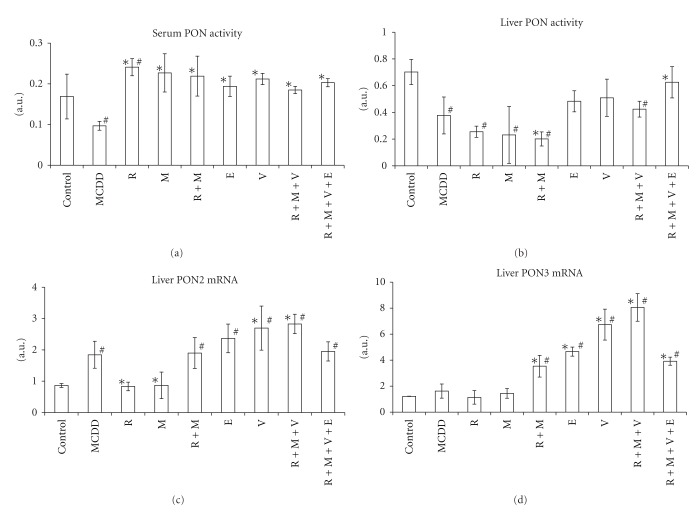
Paraoxonase (PON) activity in sera (a) and livers (b); PON2 (c) and PON3 (d) mRNA expression in rat experimental fatty liver model. Fatty liver in Sprague-Dawley rats was induced by methionine-choline deficient diet (MCDD) for 9 weeks. Rats were treated for other 6 weeks with rosiglitazone (R); metformin (M); rosiglitazone + metformin combination (R+M); ezetimibe (E); valsartan (V); rosiglitazone + metformin + valsartan in combination (R+M+V) or rosiglitazone + metformin + valsartan + ezetimibe (R+M+V+E). “^#^” indicates *P* < 0.05 versus control group (fed by standard rat chow). “*” indicates *P* < 0.05 versus MCDD group.

**Figure 3:**
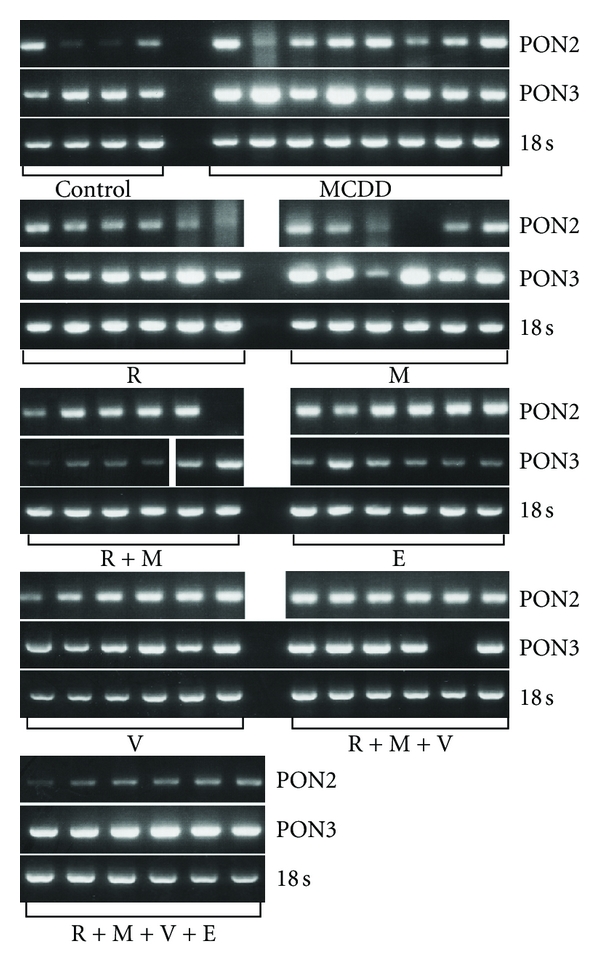
PON2 and PON3 mRNA expression in rat fatty liver experimental model. Fatty liver in Sprague-Dawley rats was induced by methionine-choline-deficient diet (MCDD) for 9 weeks. Rats were treated for other 6 weeks with rosiglitazone (R); metformin (M); rosiglitazone + metformin in combination (R+M); ezetimibe (E); valsartan (V); rosiglitazone + metformin + valsartan in combination (R+M+V) or rosiglitazone + metformin + valsartan + ezetimibe (R+M+V+E). 18 s ribosomal RNA was used for relative quantification. “^#^” indicates *P* < 0.05 versus control group (fed by standard rat chow). “*” indicates *P* < 0.05 versus MCDD group.

Rats on MCDD for 9 weeks showed increased MDA levels in serum and liver by 264% (*P* < 0.01) and 124% (*P* < 0.01), respectively (Figure 1, Table 1). PON activities in serum and liver were decreased by 43% (*P* < 0.05) and 44% (*P* < 0.01), respectively (Figures 2(a) and 2(b)). In the liver, PON3 mRNA expression was not changed, while PON2 mRNA expression increased by 113% (*P* < 0.05) (Figures 2 and 3, Table 1).

Treatment with rosiglitazone in comparison with rats on MCDD did not affect serum MDA level, while liver MDA level was lowered by 36% (*P* < 0.01), which was not significantly different from control group (Figure 1). Serum PON activity increased under rosiglitazone therapy by 43% (*P* < 0.01) in comparison with control group and by 148% in comparison with MCDD group (*P* < 0.01). Liver PON activity decreased by 68% (*P* < 0.01) under rosiglitazone treatment in comparison with control group but was not different in comparison with MCDD group (Figure 2). PON2 mRNA expression decreased by 55% (*P* < 0.01) under treatment by rosiglitazone in comparison with MCDD group, back to the level of control group. PON3 mRNA expression was not significantly different in comparison with the control and MCDD groups (Figures 2 and 3).

In rats treated with metformin, MDA levels in serum and liver were decreased by 37% (*P* < 0.01) and 58.3% (*P* < 0.01), respectively, in comparison with MCDD group. Metformin normalized MDA level in the liver, while the level in plasma was still 2.25-fold higher than in control group (*P* < 0.01) (Figure 1). Metformin treatment increased serum PON activity by 134% (*P* < 0.01) in comparison to MCDD group to the normal activity in control group. Metformin did not affect the decreased liver PON activity in comparison with MCDD group. PON2 mRNA expression decreased by 55% (*P* < 0.01) under treatment by metformin in comparison with MCDD group, back to the level of control group. PON3 mRNA expression was not significantly different in comparison with the control and MCDD groups (Figures 2 and 3).

Combined treatment with rosiglitazone and metformin in comparison with rats on MCDD decreased serum and liver MDA levels by 39% (*P* < 0.01) and 52% (*P* < 0.01), respectively. Under this treatment, plasma MDA level was still higher than control group by 120% (*P* < 0.01) and liver MDA was not different from control group. Serum PON activity increased by the combination of rosiglitazone and metformin by 126% (*P* < 0.01) in comparison with MCDD group and was not different from the control group. Liver PON activity decreased by 47% (*P* < 0.0001) and by 71% (*P* < 0.01) in comparison with MCDD and control groups, respectively. PON2 mRNA expression in the liver was not different from that in MCDD group but 120% (*P* < 0.01) above that in the control group. PON3 mRNA expression increased in the liver by 118% (*P* < 0.01) and by 190% (*P* < 0.01) in comparison with the MCDD and control groups, respectively (Figures 2 and 3).

Treatments with ezetimibe, valsartan, combined treatment with rosiglitazone-metformin-valsartan, and combined treatment with rosiglitazone-metformin-valsartan-ezetimibe decreased serum MDA levels by 27% (*P* < 0.01), 31% (*P* < 0.01), 43% (*P* < 0.01), and 42% (*P* < 0.01), respectively. None of these treatments normalized plasma MDA levels in comparison with the control group. The same treatments decreased liver MDA levels in comparison with the MCDD group by 41% (*P* < 0.01), 35% (*P* < 0.01), 57% (*P* < 0.01), and 65% (*P* < 0.01), respectively. All these treatments normalized liver MDA levels in comparison with the control group. Plasma PON activity increased significantly (*P* < 0.01) by 100%, 119%, 91%, and 110% under treatments with ezetimibe, valsartan, combined treatment with rosiglitazone-metformin-valsartan, and combined treatment with rosiglitazone-metformin-valsartan-ezetimibe, respectively. All these treatments normalized plasma PON activity towards the control group. Liver PON activity did not change under treatments with ezetimibe, valsartan, or combined treatment with rosiglitazone-metformin-valsartan in comparison with MCDD group and was still significantly lower than the activity in control group. Combined treatment with rosiglitazone-metformin-valsartan-ezetimibe increased liver PON activity by 66% in comparison with MCDD group (*P* < 0.05) which was not significantly different from control group. PON2 mRNA expression in the liver under treatment with ezetimibe or combined treatment with rosiglitazone-metformin-valsartan-ezetimibe was not significantly different in comparison with MCDD group. When compared with the control group, it was increased by 174% (*P* < 0.01) in the ezetimibe group, by 212% (*P* < 0.01) in the valsartan group, by 227% (*P* < 0.01) in the combined treatment with rosiglitazone-metformin-valsartan, and by 126% (*P* = 0.01) in the rosiglitazone-metformin-valsartan-ezetimibe group.

PON3 mRNA expression increased significantly (*P* < 0.001) in the groups ezetimibe, valsartan, rosiglitazone-metformin-valsartan, and rosiglitazone-metformin-valsartan-ezetimibe by 188%, 316%, 397%, and 142% in comparison with MCDD group and by 282%, 452%, 560%, and 221% in comparison with control group, respectively (Figures 2 and 3). 

## 5. Discussion

As was shown in previous report, there was an increased oxidative stress in the serum and liver of MCDD rat group (experimental NAFLD) [25]. In the present study, it was demonstrated that this is accompanied by decreased PON activity in the serum and liver. In the liver of MCDD rat group, PON3mRNA expression did not increase, while PON2 mRNA expression increased without increased measured enzyme activity. This may be due to oxidative inactivation of the PON2 protein. For PON1 it was shown that oxidative stress in several systems can inactivate the protein [19]. Hydroxyl radicals may be active species primarily responsible for the oxidative inactivation of PON1 in *in vivo* system [19]. Oxidized lipids were shown to inactivate both serum and hepatic PON1 [35, 36]. Free sulfhydryl groups of PON1 interact with specific oxidized lipids and by that PON1 is inactivated. Xanthine oxidase (XO) produces superoxide anions which may decrease PON1 activity. Serum XO activity was negatively correlated with PON activity [35]. In parallel, in mouse peritoneal macrophages (MPMs), oxidative stress markedly increased PON2 mRNA levels but had no effect on PON3 mRNA levels. When MPM lipid peroxides content was increased, PON2 mRNA levels were significantly higher whereas PON3 mRNA levels were similar compared with control cells [34]. *Ex vivo* as well as *in vitro* studies, showed that under oxidative stress PON2 expression and enzymatic activity increased, whereas PON3 expression did not change, while its activity decreased [34]. In the present study, the increased hepatic oxidative stress was associated with increased hepatic PON2 mRNA expression, in accordance with an other study [34]. Despite that, the hepatic PON2 activity was decreased, possibly due to oxidative inactivation of the protein. 

Oxidative stress is mediated by several oxidants which act by different mechanisms. So the next step was to examine the possibility whether polypharmacy therapy with different antioxidative mechanisms can have more potent effect to decrease the oxidative stress in NAFLD, and by that to increase the enzymatic activity of hepatic PON2. Treatments with rosiglitazone, metformin, rosiglitazone-metformin, ezetimibe, valsartan, rosiglitazone-metformin-valsartan, and rosiglitazone-metformin-valsartan-ezetimibe had all decreased oxidative stress in the liver to normal level. Liver tocopherol/MDA ratio [25], liver MDA level, and liver PON activity all were not different in the rat group which was treated by rosiglitazone-metformin-valsartan-ezetimibe in comparison with the control rat group. In the rosiglitazone-metformin-valsartan-ezetimibe rat group, serum PON activity was normalized, while serum MDA level improved significantly, but was still higher compared with the control rat group. It seems that oxidative stress sources in the serum are not limited to the liver, but can be from other sources such as skeletal muscles and adipose tissue. All these medications have direct or indirect antioxidant effect. In alloxan-induced type 1 diabetes in rats, metformin treatment led to a decrease in plasma lipid peroxidation levels compared to the nontreated group [37]. Valsartan have been shown to has antioxidative effect. Valsartan has phenolic moiety which may contribute to its free radical scavenging capacity [38]. Ezetimibe can have indirect antioxidative effect on serum LDL [28]. Moreover, in previous report, ezetimibe decreased significantly hepatic fat content, triglyceride content, and cholesterol content by 77%, 53%, and 25%, respectively [25]. Ezetimibe therapy for 2 years in patients with NAFLD improved significantly steatosis and necroinflammatory grades and ballooning and NAFLD activity scores [39]. Rosiglitazone exerted a significant vascular protective effect in hypercholesterolemic rabbits, most likely by attenuation of oxidative and nitrative stresses [40]. Rosiglitazone decreased O_2_^−^ production when it was incubated together with cadmium or zinc [41]. Moreover, rosiglitazone upregulates the mRNA and protein expression of PON2 [42]. Rosiglitazone treatment in rats with metabolic syndrome decreased hepatic lipid peroxidation and increased hepatic paraoxonase activity [43].

In parallel with the decreased oxidative stress, PON activity in the serum was normalized in all treatment groups and increased in rosiglitazone group. Van Wijk et al. reported that rosiglitazone reduced fasting plasma peroxides and increased fasting PON-1 activity, without changing PON-1 mass in type 2 diabetic patients [44]. The only therapy that normalized the decreased activity of PON in the liver was rosiglitazone-metformin-valsartan-ezetimibe. Although PON2 and PON3 mRNA expressions were increased under treatments with rosiglitazone-metformin, ezetimibe, valsartan, rosiglitazone-metformin-valsartan, and rosiglitazone-metformin-valsartan-ezetimibe, only in the last group the PON activity was normalized in the liver. Increased PON2 activity can be due to stabilization of PON2 protein, increased translational state, or increased mRNA expression. These different mechanisms can explain the difference in PON2 activity under different treatments beyond the increased expression of mRNA PON2. The supplementation of E^0^ mice with dietary antioxidants significantly increased macrophage PON3 activity, suggesting that oxidative stress was the cause for the reduced macrophage PON3 activity [34]. Moreover, PON3 lactonase activity decreases in parallel to the extent of the oxidative stress. Antioxidants have been shown to preserve PON1 activity [35] and pomegranate juice and vitamin E have similar effect on E^0^ mice macrophage PON3 activity [34]. By pharmacologic intervention, serum and hepatic oxidative stress was decreased, protecting PON2 and PON3 protein from inactivation leading to increased serum and liver PON activities. On one side, PON activity can be increased by antioxidative therapy. On the other side, it is well documented that both PON2 and PON3 have antioxidant activity. Pretreatment of cultured aortic endothelial cells with supernatants from HeLa cells over expressing PON3 prevented the formation of mildly oxidized LDL [45]. PON2 overexpression lowers the intracellular oxidative state of HeLa cells treated with hydrogen peroxide or with oxidized phospholipids [6]. PON2 and PON3 reduced oxidative stress in macrophages from E^0^ mice [34]. PON1 reduces the content of macrophage lipid peroxides [46, 47]. It seems that PON2 and PON3 may act as potent cellular antioxidants [33, 45]. In EA.hy 926 cells, both confocal microscopy and biochemical cell fractionation demonstrated a prominent enrichment of PON2 in the nuclear envelope and the ER. Results were confirmed by microscopy of overexpressed PON2-iso1-GFP in several other cell types (SMCs, AoAFs, HeLa, HEK293T, and U2OS) [48]. Experiments performed in 3 major vascular cell types demonstrate that PON2 is capable of reducing oxidative stress [48]. In the present study, increased PON2 and PON3 activities by themselves due to the medications which were used can further decrease oxidative stress. PON3 mRNA expression was increased under treatments with rosiglitazone-metformin, ezetimibe, valsartan, rosiglitazone-metformin-valsartan, and rosiglitazone-metformin-valsartan-ezetimibe. With the potential antioxidative stress of these therapies, this can culminate in increased hepatic activity of PON3.

In the present study, combined therapy with rosiglitazone-metformin-valsartan-ezetimibe was the only treatment that normalized oxidative stress in the liver, decreased oxidative stress in the serum, normalized PON activity both in the serum and liver by increasing PON2 and PON3 mRNA expressions and/or due to higher resistance of PON2 and PON3 protein to inactivation. As previously reported, the combined therapy had greater effect to increase insulin sensitivity and to improve hepatic steatosis than monotherapy in the MCDD rat model of NAFLD [25].

So it can be deduced that combined therapy by its antioxidative effect and possibly by other direct effects increased PON2 and PON3 protein activity.

In this experimental model of NAFLD, the increase in PON2 mRNA levels may be the cell response to oxidative stress, as was shown for other cellular antioxidant enzymes [49].

To conclude, increased oxidative stress in experimental NAFLD decreased PON activity in serum and liver, despite the increased expression of PON2 mRNA in the liver. Treatment with insulin sensitizers, ezetimibe, and valsartan which have antioxidative properties by different mechanisms increased PON2 and PON3 activities which culminated in normalized oxidative stress both in serum and liver. This can be due to increased PON2 and PON3 mRNA translation and/or decreased PON inactivation due to lowered oxidative stress. Improved histological findings in these rats as was shown in previous report [25] can be due, in part, to the decreased oxidative stress.

## Figures and Tables

**Table 1:** Results of serum versus liver in rat experimental fatty liver model. Fatty liver in Sprague-Dawley rats was induced by methionine-choline-deficient diet (MCDD) for 9 weeks. Rats were treated for other 6 weeks with rosiglitazone (R); metformin (M); rosiglitazone + metformin combination (R+M); ezetimibe (E); valsartan (V); rosiglitazone + metformin + valsartan in combination (R+M+V) or rosiglitazone + metformin + valsartan + ezetimibe (R+M+V+E). Malondialdehyde (MDA), Paraoxonase (PON).

	Change by %	Serum MDA	Serum PON activity	Liver MDA	Liver PON activity	Liver PON2 mRNA	Liver PON3 mRNA
MCDD	Versus control	+264 *P* < 0.01	−43 *P* < 0.05	+124 *P* < 0.01	−44 *P* < 0.01	+113 *P* < 0.05	+33 *P* = NS
	Versus MCDD						
R	Versus control	+203 *P* < 0.01	+43 *P* = 0.01	+42 *P* = NS	−68 *P* < 0.01	−4 *P* = NS	−7 *P* = NS
	Versus MCDD	−15 *P* = NS	+148 *P* < 0.01	−37 *P* < 0.01	−40 *P* = NS	−55 *P* < 0.01	−30 *P* = NS
M	Versus control	+126 *P* < 0.01	+35 *P* = NS	−7 *P* = NS	−67 *P* < 0.01	+1 *P* = NS	+18 *P* = NS
	Versus MCDD	−37 *P* < 0.01	+134 *P* < 0.01	−58 *P* < 0.01	−39 *P* = NS	−53 *P* < 0.01	−11 *P* = NS
R+M	Versus control	+120 *P* < 0.01	+30 *P* = NS	+7 *P* = NS	−71 *P* < 0.01	+120 *P* < 0.01	+190 *P* < 0.01
	Versus MCDD	−39 *P* < 0.01	+126 *P* < 0.01	−52 *P* < 0.01	−47 *P* < 0.01	+3 *P* = NS	+118 *P* < 0.01
E	Versus control	+162 *P* < 0.01	+15 *P* = NS	+32 *P* = NS	−31 *P* < 0.01	+174 *P* < 0.01	+281 *P* < 0.01
	Versus MCDD	−27 *P* < 0.01	+100 *P* < 0.01	−41 *P* < 0.01	+28 *P* = NS	+29 *P* = 0.048	+188 *P* < 0.01
V	Versus control	+148 *P* < 0.01	+26 *P* = NS	+45 *P* = NS	−27 *P* < 0.05	+212 *P* < 0.01	+452 *P* < 0.01
	Versus MCDD	−31 *P* < 0.05	+119 *P* < 0.01	−35 *P* < 0.01	+35 *P* = NS	+47 *P* < 0.05	+316 *P* < 0.01
R+M+V	Versus control	+105 *P* < 0.01	+10 *P* = NS	−4 *P* = NS	−40 *P* < 0.01	+227 *P* < 0.01	+560 *P* < 0.01
	Versus MCDD	−43 *P* < 0.01	+91 *P* < 0.01	−57 *P* < 0.01	+12.5 *P* = NS	+54 *P* < 0.01	+397 *P* < 0.01
R+M+V+E	Versus control	+109 *P* < 0.01	+21 *P* = NS	−22 *P* = NS	−11 *P* = NS	+126 *P* < 0.01	+221 *P* < 0.01
	Versus MCDD	−42 *P* < 0.01	+110 *P* < 0.01	−65 *P* < 0.01	+66 *P* < 0.01	+6 *P* = NS	+142 *P* < 0.01
